# Delayed endovascular repair for traumatic aortic pseudoaneurysms: experience from an Asian single center

**DOI:** 10.1186/s13019-022-02078-0

**Published:** 2023-01-11

**Authors:** Yuzhou Liu, Lin Sun, Qing Wang, Bin Xiang, Huangxing Cai, Yong Xie, Muzi Li, Hua Xiang

**Affiliations:** Department of Interventional Radiology and Vascular Surgery, The First Affiliated Hospital of Hunan Normal University, Changsha, 410005 People’s Republic of China

**Keywords:** Traumatic aortic pseudoaneurysms, Endovascular repair, Delayed repair

## Abstract

**Background:**

Traumatic aortic pseudoaneurysms (PSAs) classified as grade III aortic injuries are conventionally repaired as procedural emergencies, generally within 24 h of arrival. These patients typically require adequate resuscitation and treatment of multiple traumatic injuries, which complicate optimal management strategies of aortic PSAs. This study reviews the experience of an Asian single center to evaluate the efficacy and safety of delayed (> 24 h) endovascular repair for PSAs.

**Methods:**

Twenty-seven patients with blunt aortic injury (BTAI) were brought to our institution between February 2014 and May 2020. Patients with other grades of aortic injuries (grade I, II, or IV) were excluded from the study, and the remaining patients with grade III aortic injuries were placed into the early (< 24 h) and delayed (> 24 h) groups according to the timing of repair. Medical records and follow-up computed tomography (CT) scans were reviewed to document the outcomes of the procedures. Primary outcomes included mortality and complications.

**Results:**

During this period, there were 14 patients (13 males and 1 females) with aortic PSAs, and each patient received thoracic endovascular aortic repair (TEVAR). Of these 14 patients, 1 underwent emergent TEVAR, and 13 underwent delayed repair (median 7 days, range, 3–14 days). Over a period of 8 years, the overall survival of our series was 100%. No paraplegia, stroke, ischemia of limb or other serious procedural complications were observed during the duration of follow-up.

**Conclusion:**

The experience of our center indicates that delayed repair for selected PSAs could be permissible, which enables a repair in more controlled circumstances.

**Supplementary Information:**

The online version contains supplementary material available at 10.1186/s13019-022-02078-0.

## Background

Blunt aortic injury (BTAI) is a rare but devastating clinical illness coexisting with multiple trauma injuries and remains the second leading cause of death in trauma patients [1]. Conventional surgical repair has been utilized in BTAI previously until the emergence of a minimally invasive procedure via thoracic endovascular aortic repair (TEVAR). With improved outcomes demonstrated by several reports, TEVAR rapidly replaced open repair as the standard of care for these injuries [2–4]. A grading system adopted by the Society for Vascular Surgery (SVS) divided BTAI into four grades determined by the severity of injuries to guide clinical practice. Aortic pseudoaneurysms (PSAs) are classified as grade III. Grades I, II, and IV correspond to intimal tears, intermural hematoma, and aortic rupture, respectively [4]. The SVS proposed guidelines in 2011 endorsing early TEVAR as the preferred treatment method for grade II to IV BTAI [4]. However, significantly increased description of mortality benefit by delayed repair in BTAI patients has partly transformed the treatment concept of BTAI proposed by the SVS [5–11]. It has been confirmed that intimal tears will resolve via conservative treatment and do not require repair unless the grade of injury progresses [4, 8, 9]. Patients with grade II injuries can receive delayed repair safely or may not need repair actually [9–11]. Conversely, patients with grade IV injuries are in the extremis frequently and require emergency repair. However, Patients with aortic PSAs, classified as grade III injuries, fall into a “gray area”. Currently, the optimal time of repair for traumatic aortic PSAs remains a subject of debate across clinical practice. Guidelines of the Eastern Association for the Surgery of Trauma (EAST) and SVS endorse early repair (< 24 h from injury) of these lesions regardless of size [4, 8], whereas several studies suggest that many aortic PSAs, especially small lesions, are stable and can be treated with anti-impulse therapy without requiring early repair [5, 10, 12]. Given the conflicting opinions and paucity of data, particularly from Asia, we evaluate the outcomes and safety of delayed fashion for traumatic aortic PSAs drawing on the experience of a single center in Asia.

## Methods

### Patients

**Fig. 1:**
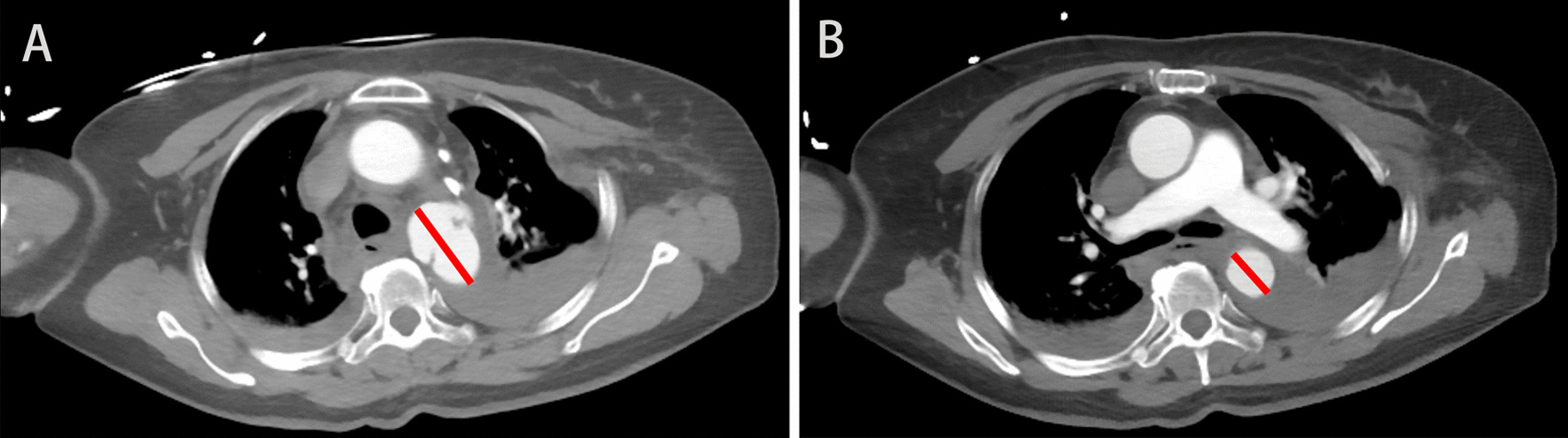
A Traumatic aortic pseudoaneurysms maximal width, B normal aortic diameter

From February 2014 to May 2020, Twenty-seven patients with BTAI were referred to Hunan Provincial People's Hospital, China. Thirteen patients with grade I, grade II injuries or grade IV injuries were excluded from the study. The demographics, the ratio of the maximum diameter of PSAs/normal aortic diameter (P/N) (the method of measurement is shown in Fig. 1), the timing and manner of aortic repair and the outcomes of the remaining 14 patients with traumatic aortic PSAs were analyzed retrospectively (Additional file 1: Table S1). Primary outcomes included mortality, ischemia of the spinal cord or limb, stroke and stent-related complications such as migration, collapse and leakage. The severity of injuries was reflected by the Injury Severity Score (ISS). According to the time interval from admission to repair, patients were placed into early (< 24 h from admission) and delayed (> 24 h after admission) repair groups.

### Initial management

**Fig. 2:**
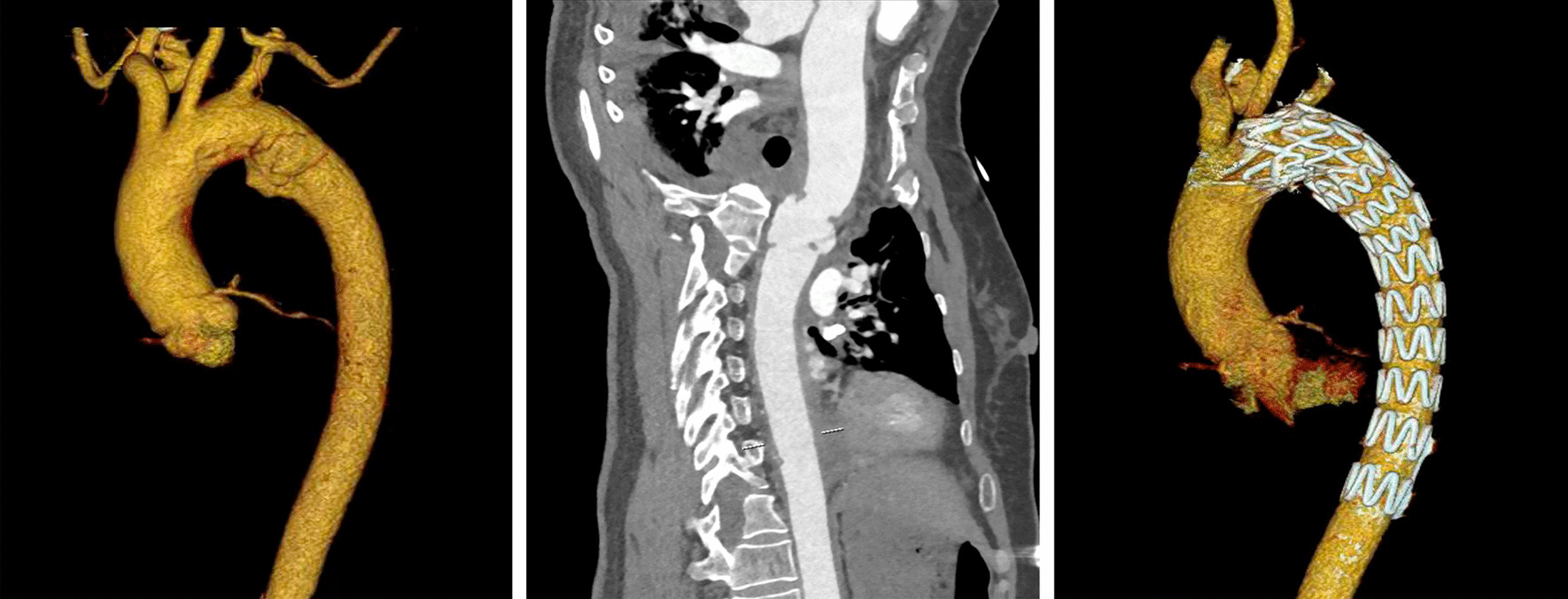
Case 14: Pseudoaneurysms with ratio of P/N = 1.79 and irregular shape were repaired early and the left subclavian artery was covered completely

**Fig. 3:**
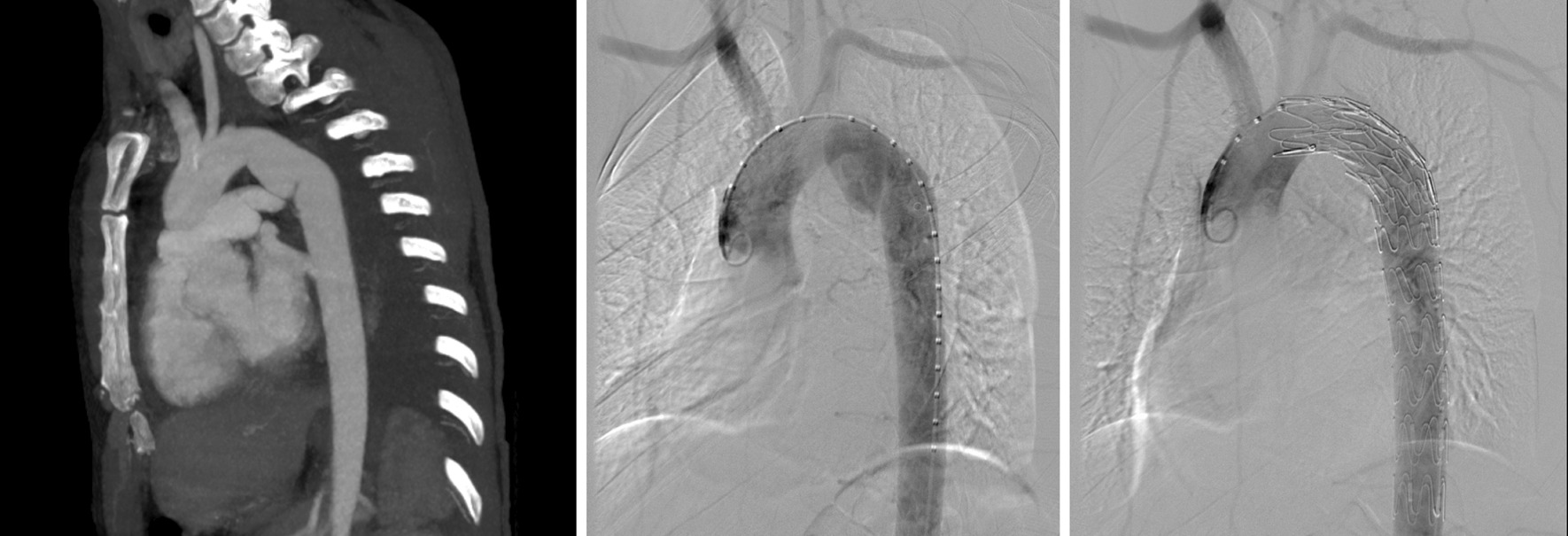
Case 9: Pseudoaneurysms with ratio of P/N = 1.67 and regular shape were received delayed repair

Three patients who experienced accidents were brought to the emergency department of our hospital directly, and the remaining 11 patients were transferred to our hospital immediately after BTAI was recognized at regional hospitals. Traumatic aortic PSAs were diagnosed by CT angiography (CTA) scans in all patients according to the grading system in SVS. When the patient was diagnosed with aortic injuries, anti-impulse therapy was performed to relieve the shear stress on the vascular wall. Goals for anti-impulse therapy consisted of achieving heart rate ≤ 80 beats/minute and systolic blood pressure (SBP) ≤ 120 mmHg. The manner and timing of management for PSAs were determined by an interventional vascular surgeon based on the radiological and clinical characteristics of the patient (Figs. 2, 3). Relevant specialist physicians were invited to assess and treat the associated injuries.

### Procedure (TEVAR)

All patients received endovascular repair. Each TEVAR procedure was performed in a hybrid operating room. The distance from the proximal injury to the left subclavian artery (LSA), the maximum diameter and length of the PSAs, and the diameter of the aorta in distal and proximal sections to injuries were measured on preoperative CTA and intraoperative angiography images. Heparin (100 IU/kg) was administered to all patients except 1 patient in the early group. After local anesthesia or general anesthesia, the common femoral artery was surgically exposed, and a pig-tail catheter inserted over the wire was passed through the femoral artery to the ascending aorta for angiography. A stent-graft oversizing of 10–15% was selected. The aortic stent-graft system inserted over the stiff wire was transported to the proximal landing zone. Then, the stent graft was deployed under fluoroscopy when a heart rate ≤ 80 beats/min and systolic blood pressure ≤ 100 mmHg were reached. If the distance from the proximal injury to the LSA was ≥ 15 mm, the graft was deployed distal to the orifice of the LSA. Conversely, partial or complete coverage of the LSA was performed to obtain adequate graft fixation. Aortography was performed repeatedly after the stent graft was deployed to ensure complete exclusion of the PSAs and correct flow of blood through the prosthesis without leakage (Figs. 2, 3).

### Follow-up

Patients were admitted to the intensive care unit (ICU) or interventional vascular surgery ward after procedures to monitor vital signs. CTA scans were performed on the patient to determine the position of the stent approximately 5 days after the procedures. Patients were followed up in the outpatient department 3, 6 and 12 months and annually thereafter postoperatively, and CTA scans were performed to monitor the aorta and graft.

## Results

TEVAR procedures were performed in all patients successfully as suitable anatomic conditions. One patient received early TEVAR, and 13 patients had initial observation. The time until definitive therapy in the delayed group ranged from 3 to 14 days.

All patients survived the TEVAR procedures without any intervention of additional surgery or conversion to open repair. We deployed Ankura (Lifetech, Shenzhen, China) in 10 cases, Hercules (Microport, Shanghai, China) in 4 cases, and Talent (Medtronic, Minneapolis, USA) in 1 case. Angiography after stent implantation immediately and CTA scans approximately 5 days after procedures showed that PSAs were excluded completely without stent migration, collapse and leakage. The LSA was covered completely in 2 patients and partially covered in 1 patient. None of the patients developed steal syndrome or other symptoms of ischemia requiring LSA reconstruction. Femoral artery thrombectomy was performed emergently in 1 patient with right femoral artery thromboembolism on the first day after TEVAR. Two patients with paraplegia prior to the TEVAR were attributed to spinal cord injuries rather than a surgical complication.

All patients survived the duration of follow-up, ranging from 6 to 97 months, and no graft-related complications, such as leakage, migration or collapse, were observed in subsequent CTA scans. Moreover, no patient in our cohort experienced serious procedural complications after TEVAR, such as paraplegia and stroke, during the follow-up period.

## Discussion

Patients with BTAI often present with significant associated injuries that should be considered when determining the timing of repair, which clouds the optimal management strategies of BTAI. Considering the risk of subsequent aortic rupture, the SVS guidelines recommend early repair [4]. However, several current large-sample studies have demonstrated that delayed intervention is associated with lower mortality than early intervention [6, 7, 13]. Nevertheless, two of the three studies did not provide information about a spectrum of BTAI [6, 7], and another one did not classify patients by grade of aortic injuries [13]. Therefore, these conclusions could not be well generalized to high-grade injuries. Traumatic PSAs are considered to be unstable lesions conventionally, and balancing the poor results of early repair with the risk of rupture remains challenging [8]. Early repair is still the prevalent management for traumatic aortic PSAs. We report the encouraging results of delayed repair for PSAs at a high-level trauma center in Asia over the past 8 years, which shows that selected traumatic aortic PSAs could be delayed safely.

Guidelines from EAST indicated that the risk of paraplegia and death with delayed intervention was significantly lower when compared with early repair [8]. Some authors argued that selection bias may be responsible for this discrepancy, more severe injuries occurred in the early group and thus have a higher overall mortality than the delay group, whereas some large-sample studies have demonstrated that the decreased overall mortality relationship with delay group was present regardless of the related injuries [6, 7]. The reason for this mortality benefit is unclear. One possible explanation is that more life-threatening injuries can be managed in a timely manner. Meanwhile, several studies found that the majority of in-hospital mortality in patients with BTAI was associated with coexisting injuries rather than aortic injuries [14–16]. These conclusions are consistent with our report. There were no aortic-related deaths in our series, which indicates that early repair of these lesions may not be mandatory. Another explanation is that delayed repair allows appropriate fluid resuscitation and stabilization for the patient. Appropriate fluid resuscitation increases spinal cord perfusion pressure (SCPP), which is thought to be correlated with a lower rate of paraplegia in the delayed group. In addition, patients with PSAs typically have associated injuries. Heparinization during early TEVAR poses a risk for worsening concurrent hemorrhagic injuries, while delayed repair allows the hemorrhagic injuries to be stabilized to reduce the risk of bleeding [17]. Therefore, it is evident that deliberate, delayed repair could be a valid approach in selected BTAI patients.

Traumatic aortic PSAs are not inherently unstable. Owing to the better outcomes of delayed repair, the distinction of unstable PSAs requiring early repair versus low-risk lesions suitable for delayed repair could improve outcomes for PSAs patients. Harris et al. identified that a ratio of P/N > 1.4 and a mediastinal hematoma > 10 mm were effective predictors for early aortic rupture [12]. Rabin et al. recommend emergency repair only for large PSAs (more than 50% circumference) with large left-sided hemothorax, pseudocoarctation, or mediastinal hematoma with mass effect, while the remaining PSAs could be safely observed before repair [10]. Unfortunately, there were no sufficient data in our report to identify any effective factors that would indicate which PSAs do not require early repair. In our experience, PSAs that require early repair were infrequent. Among the 14 patients in our center, given the risk of early rupture in one lesion with irregular shape and large size (the P/N ratio of 1.79), early repair was performed only in this patient (Fig. 2). The remaining 13 lesions presented with a regular shape, which we considered to be an important characterization of the stable PSAs, although some lesions had a P/N ratio > 1.4 slightly (Additional file 1: Table S1), this could not change our opinion that these were stable lesions. Therefore, we performed delayed repair in these 13 patients. More risk parameters for early aortic rupture need to be identified to accurately select lesions appropriate for delayed repair.

In a study by Smeds et al., 16 PSAs patients received a watchful waiting strategy during a 10-year period (from 2004 to 2014). Among these 16 patients, 3 patients died of other injuries before repair, and 13 patients underwent delayed TEVAR successfully. No aortic-related deaths occurred in this series [5]. Our practice validated the safety and efficacy of delayed repair strategies for selected PSAs. In our center, these patients initially received anti-impulse therapy with β-blockers and monitoring pulse and blood pressure. TEVAR was performed after these patients were sufficiently resuscitated and the associated injuries were stabilized. During a mean follow-up of 39 months, no aortic-related complications or deaths were found. These conclusions contribute to an increasing body of evidence that challenge BTAI management guidelines that support early repair of all PSAs.

Limitations of our study are related to the single-center, the small sample size and the retrospective nature. Selection bias undoubtedly exists in our study. Despite the above limitations, the outcomes of our center represent which lesions are stable and can be observed safely before definitive intervention.

## Conclusion

The experience of our center indicates that delayed repair for selected PSAs could be permissible, which enables a repair in more controlled circumstances. Adjunctive medical therapy plays a central role in these patients. Emerging study on PSAs has challenged the guidelines that endorse early repair of all PSAs. Further research is required to define more risk parameters to identify unstable PSAs that require early intervention.

## Supplementary Information

**Additional file 1: Table S1**. Endovascular repair of traumatic aortic pseudoaneurysms. Description of data: The table includes age (years), gender, the ratio of P/A, mechanism (type of accident), time to procedure (days), device, complications, major associated injury, ISS and duration of follow-up (months).

## Data Availability

All data generated or analysed during this study are included in this published article and its supplementary information files.

## Abbreviations

PSAs: Pseudoaneurysms
BTAI: Blunt aortic injury
CT: Computed tomography
CTA: Computed tomography angiography
SVS: Society for Vascular Surgery
TEVAR: Thoracic endovascular aortic repair
EAST: Eastern Association for the Surgery of Trauma
P/N: The maximum diameter of Pseudoaneurysms/normal aortic diameter
ISS: Injury Severity Score
SBP: Systolic blood pressure
LSA: Left subclavian artery
ICU: Intensive care unit
SCPP: Spinal cord perfusion pressure
